# Deep learning augmented ECG analysis to identify biomarker-defined myocardial injury

**DOI:** 10.1038/s41598-023-29989-9

**Published:** 2023-02-27

**Authors:** Gunvant R. Chaudhari, Jacob J. Mayfield, Joshua P. Barrios, Sean Abreau, Robert Avram, Jeffrey E. Olgin, Geoffrey H. Tison

**Affiliations:** 1 Department of Medicine, University of California, 555 Mission Bay Blvd South Box 3120, San Francisco, CA 94158 USA; 2 Division of Cardiology, University of Washington, Seattle, USA; 3 Division of Cardiology, University of California, San Francisco, USA; 4 Cardiovascular Research Institute, University of California, San Francisco, USA; 5 Bakar Institute of Computational Health Sciences, University of California, San Francisco, USA

**Keywords:** Machine learning, Cardiology, Health care

## Abstract

Chest pain is a common clinical complaint for which myocardial injury is the primary concern and is associated with significant morbidity and mortality. To aid providers’ decision-making, we aimed to analyze the electrocardiogram (ECG) using a deep convolutional neural network (CNN) to predict serum troponin I (TnI) from ECGs. We developed a CNN using 64,728 ECGs from 32,479 patients who underwent ECG within 2 h prior to a serum TnI laboratory result at the University of California, San Francisco (UCSF). In our primary analysis, we classified patients into groups of TnI < 0.02 or ≥ 0.02 µg/L using 12-lead ECGs. This was repeated with an alternative threshold of 1.0 µg/L and with single-lead ECG inputs. We also performed multiclass prediction for a set of serum troponin ranges. Finally, we tested the CNN in a cohort of patients selected for coronary angiography, including 3038 ECGs from 672 patients. Cohort patients were 49.0% female, 42.8% white, and 59.3% (19,283) never had a positive TnI value (≥ 0.02 µg/L). CNNs accurately predicted elevated TnI, both at a threshold of 0.02 µg/L (AUC = 0.783, 95% CI 0.780–0.786) and at a threshold of 1.0 µg/L (AUC = 0.802, 0.795–0.809). Models using single-lead ECG data achieved significantly lower accuracy, with AUCs ranging from 0.740 to 0.773 with variation by lead. Accuracy of the multi-class model was lower for intermediate TnI value-ranges. Our models performed similarly on the cohort of patients who underwent coronary angiography. Biomarker-defined myocardial injury can be predicted by CNNs from 12-lead and single-lead ECGs.

## Introduction

Chest pain is the chief complaint for more than 6.5 million Emergency Department (ED) visits in the United States each year, generating more than 28 million electrocardiograms (ECGs)^1^. Of primary concern for any chest pain complaint is myocardial injury, which includes myocardial infarction (MI), and is associated with significant morbidity and mortality. ECG and serum troponin measurement are the two central diagnostic and screening tests for chest pain evaluation and are core components of the American Heart Association Guidelines^2–6^. Upon cardiomyocyte damage, troponin is released into the bloodstream, and serum troponin is a specific biomarker for myocardial injury^7^. Despite ECG being the most commonly performed cardiac diagnostic test^1^, ECG interpretation can vary in accuracy depending on reader skill, and frequently requires historical and clinical context to be useful^2,8^. Though the annual cost of evaluation and treatment of chest pain is estimated to be between $5 and 10 billion^1,4,9–11^, only 5.5% of patients seen in the ED setting for this complaint are ultimately found to have an acute medical illness requiring hospitalization, while more than half are diagnosed with non-cardiac chest pain^12^.

Machine learning may facilitate both timely recognition of myocardial injury and reduction in the cost of chest pain evaluation. Recent advances in the field have demonstrated that neural network-based ECG analysis is capable of detecting patterns with important clinical and physiologic correlates that may not be as easily discernable by humans. Examples include arrhythmia prediction^13^, estimation of left ventricular mass and mitral annular e′ velocity^14^, prediction of serum potassium concentration using single-lead ECG^15^, detection of asymptomatic left ventricular dysfunction^16^, prediction of atrial fibrillation based on sinus rhythm ECG^17^, and screening for and tracking of chronic diseases such as pulmonary hypertension or hypertrophic cardiomyopathy^18,19^. ECGs of patients presenting with myocardial injury likely also contain similar patterns, including signals of myocardial damage that may be undetectable to clinician readers, but which may contain information related to biomarker-defined myocardial injury.

The need to develop extensive, hand-crafted feature extraction limited earlier attempts to apply machine learning to ECG interpretation, both because the process is labor intensive and because it relies on the same heuristics used by human readers. Deep convolutional neural networks (CNN) eliminate this obstacle by automating and integrating feature extraction^20^, and have shown significant promise in various applications^21–23^. A deep learning approach to ECG analysis allows for inclusion of features that may be visually imperceptible or dependent on complex patterns across multiple leads. To our knowledge there have been no published efforts to apply deep learning to predict biomarker-defined myocardial injury.

Given the need to develop safer, more cost-effective systems of care for patients with chest pain, we aimed to develop a CNN to identify myocardial injury as evidenced by elevated serum troponin I (TnI) using the 12-lead ECG. We hypothesized that deep learning analysis of ECG data would enable prediction of elevated serum TnI. To test our hypothesis, we used data extracted from the University of California, San Francisco (UCSF) electronic medical record (EMR) to train, validate, and test deep learning algorithms to predict TnI values using various thresholds. We then asked if CNNs could be trained to make the same inferences using only single-lead ECG data. This work lays the foundation for the development of rapid, cost-effective, and accurate chest pain evaluation pathways utilizing deep learning augmented ECG analysis.

## Methods

### Population and oversight

All patients who received an ECG at UCSF between January 31, 2005 and January 7, 2016 were eligible for inclusion. Any ECG from the study period was included if it was obtained within 2-h prior to a serum TnI test result. In a sensitivity analysis, we also performed this analysis in a sub-cohort of patients who underwent coronary angiography at UCSF, had an ECG obtained within 2 days prior to the angiogram procedure, and had a serum TnI test result within 4 h of the ECG recording. This study was reviewed and approved by the Institutional Review Board of the University of California, San Francisco who exempted informed consent. All research was performed in accordance with relevant guidelines/regulations.

### Outcomes

Our primary goal was to train a CNN to predict positive TnI values, defined as greater than or equal to 0.02 µg/L, utilizing standard 10-s 12-lead ECG voltages as inputs. An alternative TnI threshold of 1.0 µg/L was also evaluated. In addition to standard testing and validation of this model, we also used the trained model to make predictions on the subset of patients who underwent coronary angiography. Given the numerous possible etiologies for positive TnI values, ranging from cardiac ischemia to myocarditis, patients who underwent angiography were presumed a-priori to represent a more homogenous subset more likely to have had acute coronary syndrome (ACS)-related positive TnI. Similar algorithm performance in this sub-cohort would demonstrate predicting troponin from ECG remains consistent in ACS-likely populations. We also evaluated performance of CNNs trained on single-lead ECGs for binary TnI classification. Finally, we trained a separate CNN algorithm to classify patients into categories of predicted TnI ranges: < 0.02 µg/L, ≥ 0.02 µg/L to < 0.5 µg/L, ≥ 0.5 µg/L to < 2 µg/L, and ≥ 2 µg/L.

### Deep learning model development and training

**Figure 1:**
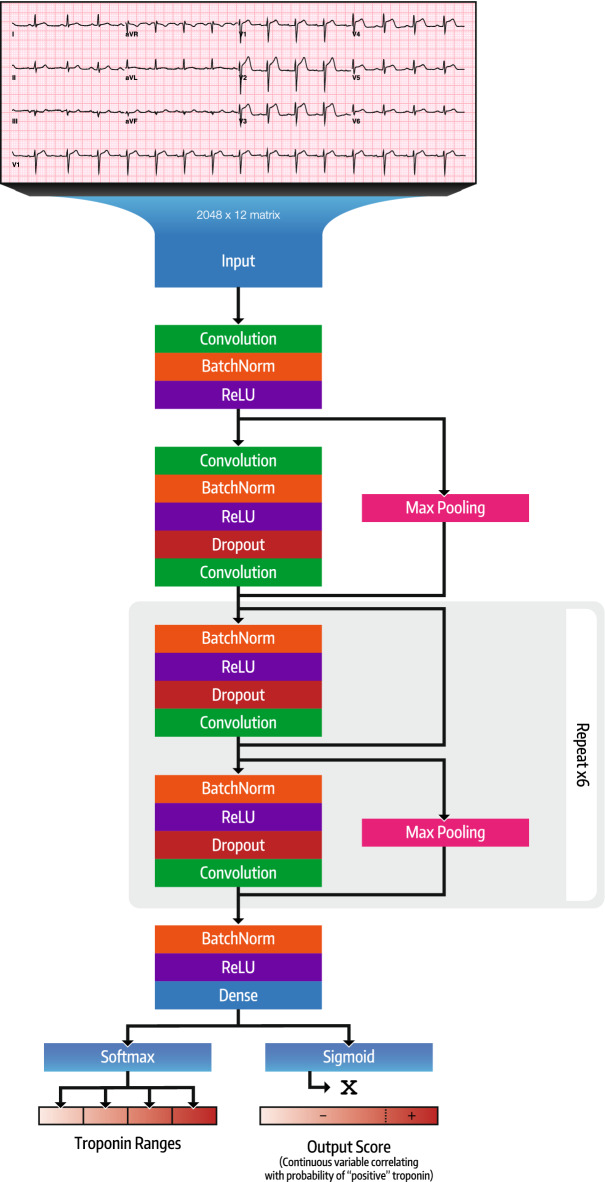
Deep convolutional neural network architecture. Boxes represent layers of the neural network; box labels indicate layer type. A sigmoid activation was used for binary classification, while a softmax activation was used for multi-class classification.

We implemented a CNN (Fig. 1) to accept input from standard 10 s 12-lead ECG data sampled at 250 Hz. ECG data was processed at time of acquisition by normalizing between leads and converting to millivolts. The CNN is based on a previously validated architecture^13^ and consists of 15 layers organized into 13 blocks with 1 convolution per block, along with shortcut connections between blocks. Prior to the convolution layer, each block has batch normalization, rectifier linear (ReLU) activation, and dropout with probability of 0.2. Every 2 blocks, the inputs are subsampled by a factor of 2. The convolutional layers have a filter width of 16 and initial filter count of 64 that double every 4 blocks. After the blocks, one fully connected layer of size 1024 was used prior to the output layer, which consisted of a sigmoid activation for the binary networks and a softmax activation for the multiclass network. We tested multiple network structures, including varying number of blocks between 10 and 15, changing dropout probability between 0.0 and 0.5, and changing initial filter count between 16 and 64. This network architecture was chosen for having the highest validation-dataset AUC. The CNN was implemented in python using Keras with Tensorflow (Google Inc.) backend.

For each CNN algorithm that was trained, we randomly split the ECG data into training, validation, and test datasets sized of 70%, 15%, 15% respectively; datasets contained mutually exclusive patients. To improve the robustness and repeatability of performance estimates, we trained 10 versions of all algorithms on 10 random splits of the data to obtain mean statistical values ± one standard deviation, unless otherwise specified. To improve generalizability, data augmentation was performed by dilating or contracting the raw ECG data (matrix size 2500 × 12) by a factor between 0.85 and 1.15 along the time axis and by selecting the fixed-sized 2048 × 12 sample input matrix randomly from within the resulting ECG matrix. The network was trained with normally initialized layer weights as previously described^24^. We used Adam optimizer^25^ with default parameters and a batch size of 64. After a grid search on learning rates ranging from 1 × 10^–3^ to 1 × 10^–5^, a learning rate of 1e-3 was chosen, with a reduction by a factor of 10 for every two epochs that the validation set loss did not improve. All training was done on one NVIDIA GeForce GTX 1080 Ti graphical processing unit. The model consisted of 15,427,457 trainable parameters and took about 71 s to train per epoch on a consumer grade GPU. Peak model performance on the validation set was achieved at epoch 19.5 on average.

In the sensitivity analysis whereby the (binary classification) CNN algorithm was deployed on the coronary angiogram sub-cohort, patients in the coronary angiogram sub-cohort were removed from the CNN training dataset and the CNN was re-trained on the remaining dataset and deployed on the coronary angiogram sub-cohort.

### Saliency ma*ps*

In order to better understand what parts of the ECG contribute the most to the CNN’s predictions of myocardial injury, we generated saliency maps for the CNN trained at cutoff 0.02 using guided backpropagation^26^ with the iNNvestigate tool (v1.0.9)^27^. Three representative positive class samples and one representative negative class sample were selected. Values below 0 were set to 0 and each map was separately scaled to between 0 and 1. After visual review of each map, the most active 4.1 s blocks from leads I, V2, V3, and V5 were selected. We also present visualizations of filters-outputs from specific CNN layers by mapping filter values to a color schema.

### Statistical analysis

In all cases, the performance of our models was assessed by sensitivity, specificity, F_1_ score, and area under the curve (AUC) of the receiver operating characteristic (ROC) curve values. *P* values were calculated from χ^2^ test for categorical variables and 1-way analysis of variance testing for continuous variables.

## Results

A total of 64,728 ECGs from 32,479 unique patients met inclusion criteria for the primary analysis (Table 1), providing a mean of 1.99 ECGs per patient (SD ± 2.38). Seventy-seven ECGs were excluded due to uninterpretable data (e.g., multiple leads with signal variability of less than 0.1 mV or one or more leads with aberrantly large signal over 100 mV). The majority of ECGs (57.2%, n = 27,693) were associated with TnI value < 0.02 µg/L. Cohort patients were 49.0% female, 42.8% white, and 59.3% (19,283) never had a positive TnI value (≥ 0.02 µg/L). The median ages of patients with all negative troponins vs. patients with one or more troponin ≥ 0.02 µg/L were 60 and 68, respectively.

**Table 1:** Patient-level study demographics stratified by presence of at least one abnormal serum troponin I value (defined as ≥ 0.02 µg/L).

Serum troponin I concentration				
	< 0.02 µg/L	≥ 0.02 µg/L	*p* value	Total
Sex—no. (%)				
Female	9956 (62.6)	5947 (37.4)	< 0.001*	15,903 (49.0)
Male	9321 (56.3)	7245 (43.7)		16,566 (51.0)
Race/ethnicity—no. (%)				
White	8489 (61.0)	5418 (39.0)	< 0.001*	13,907 (42.8)
Black	1973 (56.9)	1496 (43.1)	0.003*	3469 (10.7)
Asian/Pacific Islander	2058 (59.6)	1394 (40.4)	0.768	3452 (10.6)
Hispanic	1232 (66.8)	611 (33.2)	< 0.001*	1843 (5.7)
Native American	33 (60.0)	22 (40.0)	0.924	55 (0.2)
Other/unknown	5498 (56.4)	4255 (43.6)	< 0.001*	9753 (30)
Age				
Mean (± SD)	59.8 (16.9)	66.9 (16.6)	< 0.001*	62.6 (17.2)
Median	60	68		63
Total—no. (%)	19,283 (59.3)	13,196 (40.6)		32,479

± SD indicates standard deviation. *P* values calculated from χ^2^ test for categorical variables and 1-way analysis of variance testing for continuous variables.

*Indicates statistical significance.

### Predicting elevated TnI using 12-lead ECG

**Figure 2:**
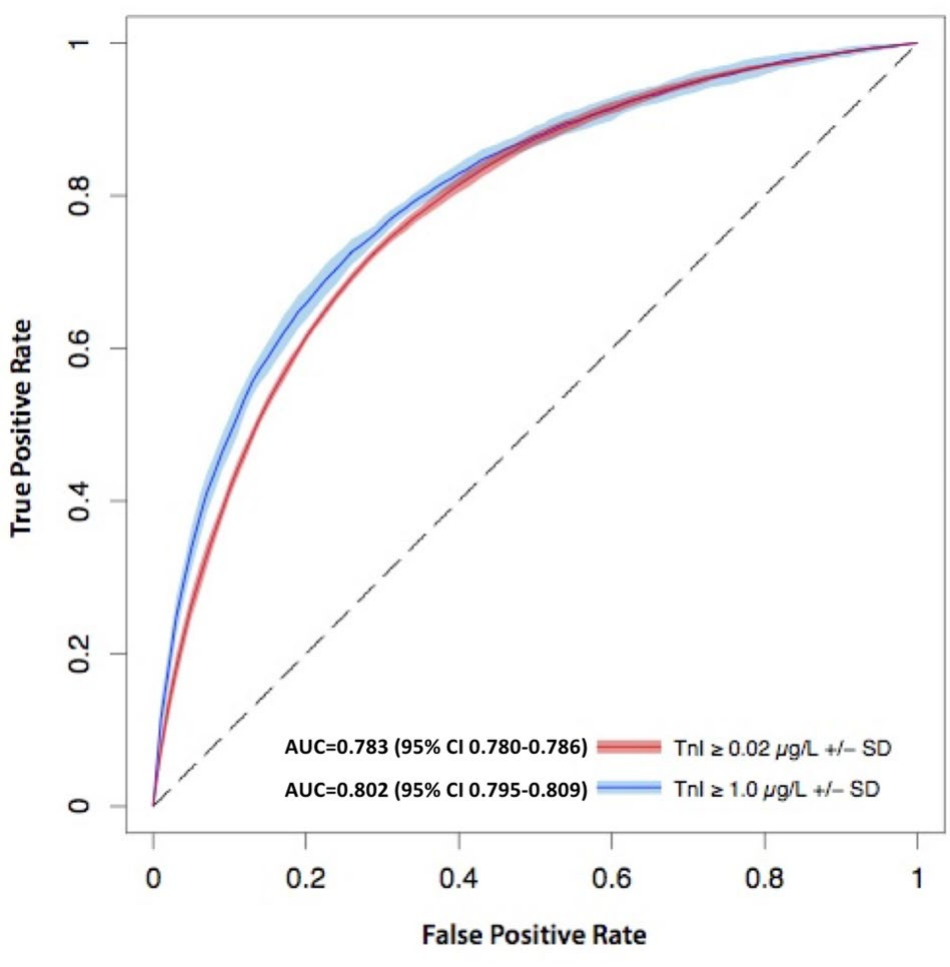
Receiver operating characteristic curves for deep neural network discrimination of Troponin I at 0.02 µg/L and 1.0 µg/L thresholds in the test dataset using 12-lead ECG. Dark lines and shaded regions indicate mean and std, respectively, across 10 models trained on different splits of the data. N = 32,479 patients.

CNN algorithms trained to discriminate 12-lead ECGs associated with TnI < 0.02 µg/L vs. ≥ 0.02 µg/L achieved an overall AUC of 0.783 (95% CI 0.780–0.786) (Fig. 2), averaged across 10 algorithms. At an operating threshold where sensitivity for a “positive” TnI ≥ 0.02 µg/L was 80.0%, the CNN achieved a specificity of 61.9% (95% CI 60.9–63.0%), a negative predictive value (NPV) of 80.4% (95% CI 80.1–80.8%), a negative likelihood ratio (LR) of 0.32 (95% CI 0.32–0.33), a positive LR of 2.1 (95% CI 2.0–2.2), and an accuracy of 69.7% (95% CI 69.0–70.3%). At an operating threshold where specificity for a “positive” TnI ≥ 0.02 µg/L was 80.0%, the CNN achieved a sensitivity of 61.4% (95% CI 60.9–61.9%), a positive predictive value (PPV) of 69.8% (95% CI 69.2%-70.4%), a positive LR of 3.1 (95% CI 3.1–3.1), a negative LR of 0.48 (95% CI 0.48–0.49), and an accuracy of 72.1% (95% CI 71.9–72.2%). We also developed CNN algorithms that were trained to discriminate 12-lead ECGs associated with TnI < 1.0 µg/L and ≥ 1.0 µg/L, that achieved an AUC of 0.802 (95% CI 0.795–0.809) (Fig. 2). For this TnI threshold, at sensitivity for a “positive” TnI ≥ 1.0 µg/L of 80.0%, the CNN achieved a specificity of 64.3% (95% CI 63.0–65.5%), and at a specificity for a “positive” TnI ≥ 1.0 µg/L of 80.0%, the CNN achieved a sensitivity of 65.9% (95% CI 64.3–67.5%).

### Classifying multiple TnI ranges using 12-lead ECG

**Figure 3:**
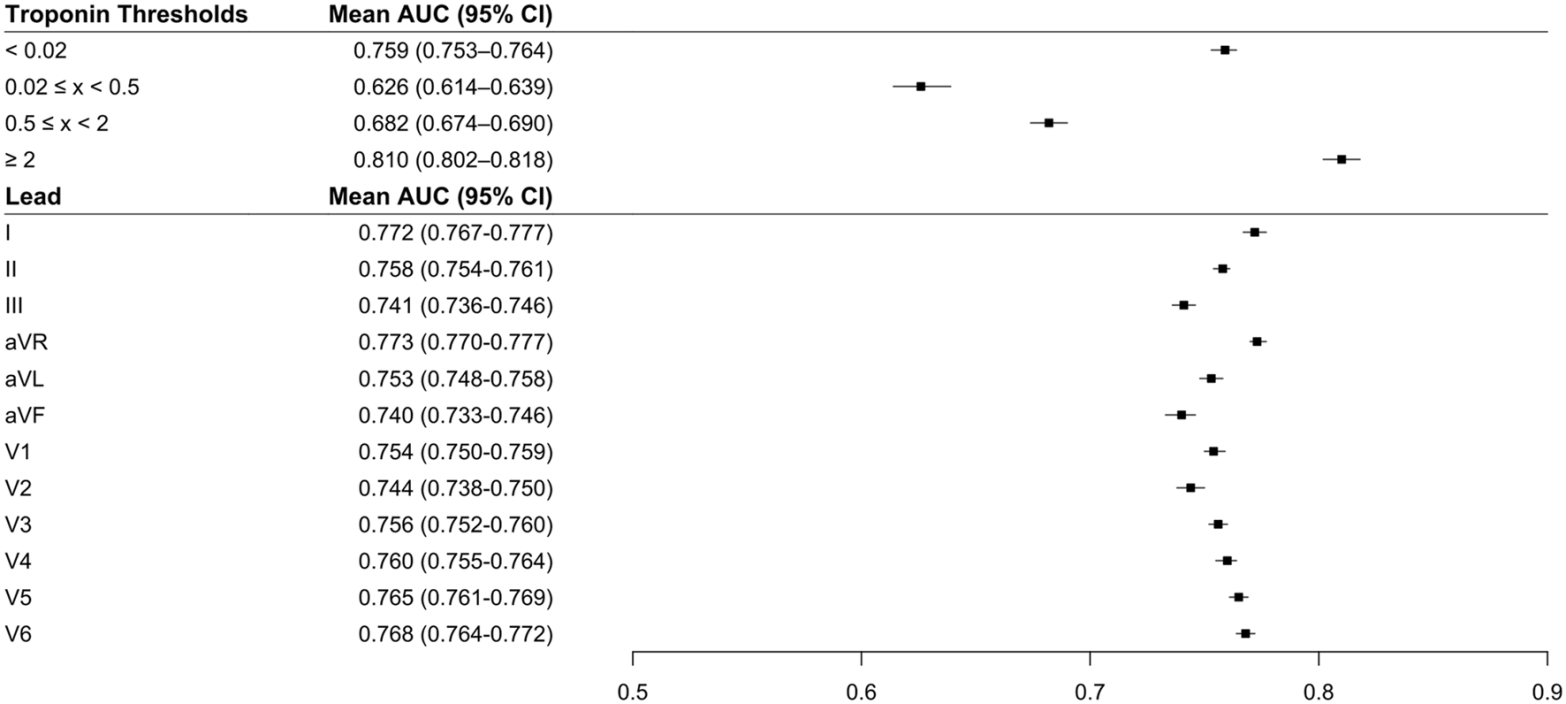
AUCs for multi-class discrimination of serum troponin ranges (upper left) and results of using single-lead ECG inputs (lower left). Right panel: Plot showing mean AUC and 95% CI for each troponin threshold or lead. Boxes and lines indicate mean and 95% CI, respectively, across 10 models trained on different splits of the data. Single lead models were trained using data from each of the 12 leads to discriminate between TnI < 0.02 µg/L and ≥ 0.02 µg/L. N = 32,479 patients.

We then trained separate CNN algorithms to discriminate several categories of TnI value-ranges based on the 12-lead ECG. For these multiclass algorithms, the CNN performed the best on high and low TnI ranges. By range of TnI value, AUCs were: 0.759 (95% CI 0.753–0.764) for TnI < 0.02 µg/L, 0.626 (95% CI 0.614–0.639) for ≥ 0.02 µg/L and < 0.5 µg/L, 0.682 (95% CI 0.674–0.690) for ≥ 0.5 µg/L and < 2 µg/L, and 0.810 (95% CI 0.802–0.818) for ≥ 2 µg/L (Fig. 3). In sensitivity analyses, multi-class CNN performance was similar when the low TnI threshold was set at 0.04 µg/L.

### Predicting elevated TnI using single-lead ECG

Since remotely monitored electrocardiographic devices—such as ambulatory monitoring, telemetry or even smartwatch ECGs—increasingly rely on fewer than the full 12-leads of a standard electrocardiogram, we examined the ability to build CNNs to identify a positive troponin using only single ECGs leads as inputs. Separate CNN algorithms were trained using ECG data from each of the 12 leads to discriminate a TnI < 0.02 µg/L and ≥ 0.02 µg/L. AUCs for the single-lead CNNs were significantly lower than AUC for the 12-lead model (AUC = 0.783), ranging from 0.773 (lead aVR) to 0.740 (lead aVF) (Fig. 3).

### Performance of CNN in a sub-cohort of patients referred for coronary angiography

In a sensitivity analysis, we used the CNN trained on the full cohort to discriminate TnI < 0.02 µg/L from ≥ 0.02 µg/L and examined its performance on the subset of patients who underwent a coronary angiogram at UCSF. Six hundred and seventy-two patients met criteria for inclusion in the coronary angiography sub-cohort, contributing a total of 3038 eligible ECGs (see “Methods” section). The sub-cohort was 32.3% (217) female, 35.0% (233) white and average age was 66.2 years (SD 13.1). ECGs paired with TnI values < 0.02 µg/L constituted 19.0% (578) of this cohort. The performance of the trained CNN algorithm in this sub-cohort (AUC = 0.766) was similar to that of the CNN in the revised test dataset (AUC = 0.777) after removing sub-cohort patients from the training data (see “Methods” section).

### Saliency map analysis of CNN

**Figure 4:**
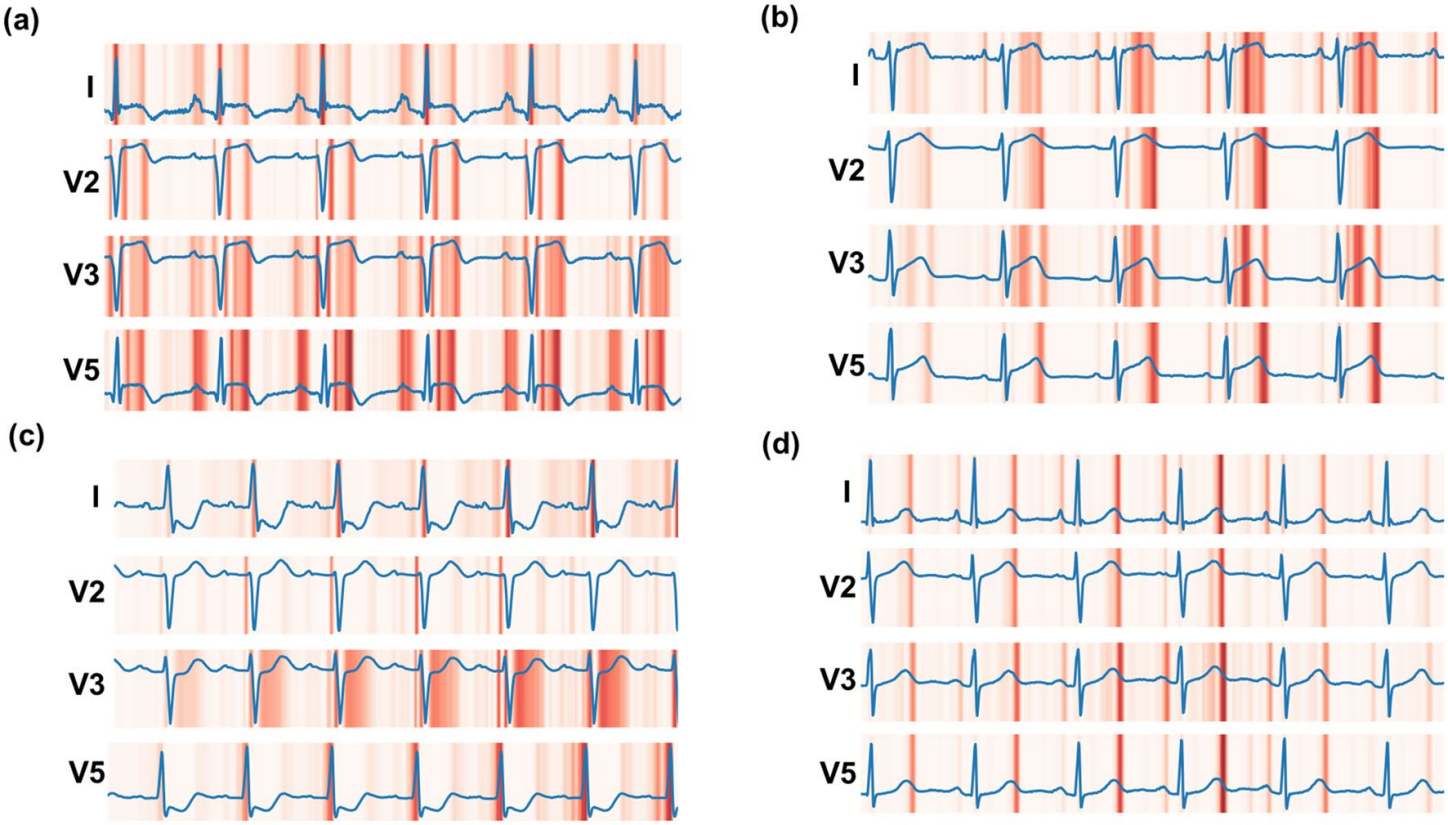
Representative saliency maps for the troponin discrimination network trained at cutoff of 0.02. Maps for leads I, V2, V3, and V5 are shown over 4.1 s of ECG acquisition. (**a**) Positive class (TnI of 50). (**b**) Positive class (TnI of 23). (**c**) Positive class (TnI of 7). (**d**) Negative class (TnI of 0).

We generated saliency maps to visualize which ECG regions were of greatest importance to the CNN for its troponin predictions. For the CNN trained to discriminate TnI < 0.02 µg/L from ≥ 0.02 µg/L, saliency maps for the troponin-positive class (Fig. 4a–c, Supplementary Fig. 2a–c) showed increased importance of ST segments and T waves, and to a lesser degree Q wave and P regions. Saliency maps for the negative-troponin class (Fig. 4d, Supplementary Fig. 2d) were more homogenous and fewer ECG regions of importance were highlighted, which included terminal T-wave regions and to a lesser degree the terminal P wave. Convolutional filter outputs (Supplementary Fig. 1) were much more complex for the positive class than for the negative class, suggesting that the CNN identified more complex features associated with elevated TnI ECGs compared to TnI negative ECGs. Convolutional filters of elevated TnI ECGs showed relatively greater activation in the ST segment and P wave/PR interval regions of the ECG (Supplementary Fig. 1).

## Discussion

This study demonstrates that biomarker-defined myocardial injury can be predicted from raw 12-lead ECG data with a CNN. CNN-enabled analysis of the ECG—a rapid, inexpensive, and ubiquitous test—may improve the efficacy and cost effectiveness of the diagnostic evaluation of chest pain. We also demonstrate that single-lead ECGs are capable of this task, further expanding this technique to a wider range of care settings, including remote ECG monitoring.

Our primary CNN algorithm sought to detect any elevated TnI plasma concentration based on 12-lead ECG data. Among the highly heterogeneous population of all patients receiving an ECG at a quaternary institution for any indication, CNNs performed moderately well to identify “positive” TnI at thresholds of 0.02 µg/L and 1 µg/L. These thresholds were selected based on the laboratory assay-defined upper limit of normal and common clinical practice, respectively. We found particular efficacy at an operating threshold optimized for sensitivity of 80% for TnI threshold of 0.02 µg/L. Here, the CNN achieved an NPV of 76.9% with a negative LR of 0.32, illustrating that CNN-enabled ECG analysis may be used to rule out biomarker-defined myocardial injury in low-risk patients. While human performance on this task has not been explicitly evaluated, a recent study of emergency physicians, cardiologists, and interventional cardiologists found that the average AUC for identification of ST-segment elevation ACS, ostensibly a more recognizable phenomenon, was 0.72^28^, slightly lower than the performance of most of our models.

Our work relied upon a large dataset of real-world clinical data and findings remained consistent in clinically-relevant sensitivity analyses, corroborating previous successes of machine learning models for similar tasks. Recently, Liu et al. developed a CNN approach utilizing multi-lead ECG data (n = 112) to predict clinically diagnosed anterior myocardial infarction (MI) with good results^29^. Despite limited sample size, this work demonstrated that deep learning can be applied effectively to this task with relatively low computational resource cost. Several groups have also employed machine learning techniques to predict MI using varied combinations of clinical and laboratory features as inputs^30–32^. Most recently, Than and colleagues utilized gradient boosting to predict Type 1 MI using age, sex, and paired high-sensitivity cardiac troponin as inputs, ultimately demonstrating performance characteristics exceeding those of the European Society of Cardiology 0/3-h pathway^3,30^. A major limitation of these efforts is the relatively narrow focus on MI, one type of myocardial injury. Our work is further distinguished by using a CNN to analyze the raw ECG waveform, making it possible for the CNN to identify ECG signals not readily interpreted by human readers. This work provides the basis for a tool to predict biomarker-defined generalized myocardial injury.

One of the more promising findings from our work is the performance of models trained using only single-lead ECG data. At-home single-lead and multi-lead ECG has increasingly become available to consumers in the form of internet-connected wearable devices. Our work demonstrates that CNNs could assist with triage, diagnosis and monitoring for myocardial injury in the remote monitoring setting using self-lead or multi-lead ECG remote monitoring devices, including smart watches. This may increase the accessibility of rural or under-resourced communities in which access to laboratory services is limited to myocardial injury monitoring when clinically indicated. The COVID-19 pandemic has highlighted the need to effectively triage low-risk patients away from high-volume emergency departments. This work provides support toward achieving this goal for chest pain, a common and high-consequence cause for presentation to emergency departments. A recent study by Jin et al.^33^ did also show that single-lead ECG data from the emergency department could be used to detect troponin elevation, reporting similar but slightly lower performance.

Our efforts to use a multi-class strategy to stratify patients into TnI groups based on surface ECG yielded mixed results. The model was able to classify ECGs into undetectable (< 0.02 µg/L) and significantly elevated (≥ 2 µg/L) groups with good accuracy, but midrange discrimination was poor. We hypothesize that this may be at least partially explained by greater phenotypic heterogeneity in patients with lower levels of detectable TnI. It is well-known that a variety of disease processes can yield elevated TnI^5^, however patients with plasma TnI levels above 2 µg/L may represent a more homogenous set of etiologies including acute coronary syndrome.

In order to test the performance of our primary model on a more homogenous cohort, we attempted to identify a subset of patients with higher pre-test probability for myocardial ischemia by identifying a subgroup of patients who underwent coronary angiography. This analysis demonstrated similar AUCs for the primary analysis and the angiography subgroup, which is likely partially explained by training population heterogeneity. Even in ACS, there is a spectrum of ECG changes suggestive of MI, and it is well-recognized that these changes are dynamic over the course of an infarction^34^. Further, revascularization can arrest the progression of electrical injury and sometimes results in normalization of the ECG^35^, a phenomenon which may have resulted in pairing of normalized ECGs with elevated TnI measurements in our dataset (within our 4 h window inclusion criteria). One recent study compared a deep learning algorithm to human physicians to detect angiogram-defined NSTEMI/STEMI ECGs showing that the algorithm significantly outperformed physicians, and that the algorithm performance further increased by adding TnI^36^.

We applied several “AI explainability” techniques to our trained CNN, such as saliency maps, to better demonstrate regions of the ECG that the CNN learned for itself during training as having greater importance to predict elevated TnI. More complex CNN-learned features were clearly associated with high TnI ECGs compared to normal-TnI ECGs, as evidenced by analysis of convolution filter outputs. Saliency maps highlighted some ECG regions well-understood to be associated with myocardial ischemia, such as the ST segment^5^, which would be expected and provided some degree of validation that the CNN is learning patterns from the data consistent with physiologically understood ECG-changes. What was noteworthy were the positive-troponin ECG examples that did not have visible ST segment changes but which were still highlighted by the CNN saliency maps, suggesting that the CNN may be identifying non-visible patterns in the ST segment. Furthermore, the identification of the P wave and PR regions of TnI-positive ECGs were more unexpected, as these are not typically associated with myocardial injury. These regions were consistently highlighted across multiple TnI-positive ECGs and, if replicated, may thus constitute a data-driven finding worthy of additional physiologic investigation. Findings such as these exemplify the type of analysis which may ultimately help to realize the fullest potential of “big-data” in medicine, whereby large-scale analysis by machine learning of medical data is able to identify heretofore unappreciated and physiologically-relevant signals in everyday diagnostic tests.

Our study had a number of limitations that should inform future efforts. A major barrier to training CNNs to recognize MI is the heterogeneity of disease processes that result in myocyte necrosis. Some patients with longstanding underlying conditions such as renal insufficiency may have chronically elevated TnI^5^. Similarly, acute perturbation in non-cardiac organ systems can result in Type 2 MI^36,37^. In our study, we were not able to differentiate between these disease processes given available data, nor were we able to differentiate between ECGs obtained before or after interventional therapies. To improve test characteristics and provide additional decision support, future efforts should attempt to better phenotype patients with positive TnI assays, categorizing them into those with ACS (Type 1 MI), those with demand infarction (Type 2 MI), and those with myocardial injury (e.g., myocarditis). While we studied a large cohort of patients, the single-center design may limit generalizability to other centers in the United States and globally. Additionally, MI is defined as a change in TnI of ≥ 20%^5^, but we considered only individual ECG-TnI pairs in this analysis. Finally, the observational nature of our study introduces both evident and hidden confounders. For example, the fact that a TnI assay was ordered by a provider suggests that the population has a higher-than-average pre-test probability for cardiovascular pathology. Controls in future efforts would ideally be members of the general population.

This study demonstrates that CNNs have the ability to predict biomarker-defined myocardial injury with good accuracy based on single-lead and 12-lead ECGs. The algorithm’s negative predictive value makes it particularly well-suited for triage of low-risk patients with chest pain using a simple and cost-effective ECG. Continued investigation utilizing better defined phenotypic groups is necessary to mature this paradigm for use in clinical practice.

## Supplementary Information

Supplementary Figures.

## Data Availability

The data used in this study are derived from clinical care and thus are not made publicly available due to data privacy concerns. Reasonable requests for collaboration using the data can be made from the corresponding author (geoff.tison@ucsf.edu), as feasible and permitted by the Regents of the University of California.

## Code availability

The code that supports this work is copyright of the Regents of the University of California and can be made available through license.
