# Supplementary material for: Deep learning augmented ECG analysis to identify biomarker-defined myocardial injury
Source: Sci Rep. 2023 Feb 27;13:3364. doi: 10.1038/s41598-023-29989-9 (PMC9969952; doi:10.1038/s41598-023-29989-9)

**Supplementary Figure S1:** Representative filter outputs for the troponin discrimination network trained at a cutoff of 0.02. Shown are the first convolution layer filter (n=64) outputs for (a) positive class ECG (Tn1 of 50) and (b) negative class ECG (TnI of 0). Red hues represent positive signal and blue hues represent negative signal.

**(a)**


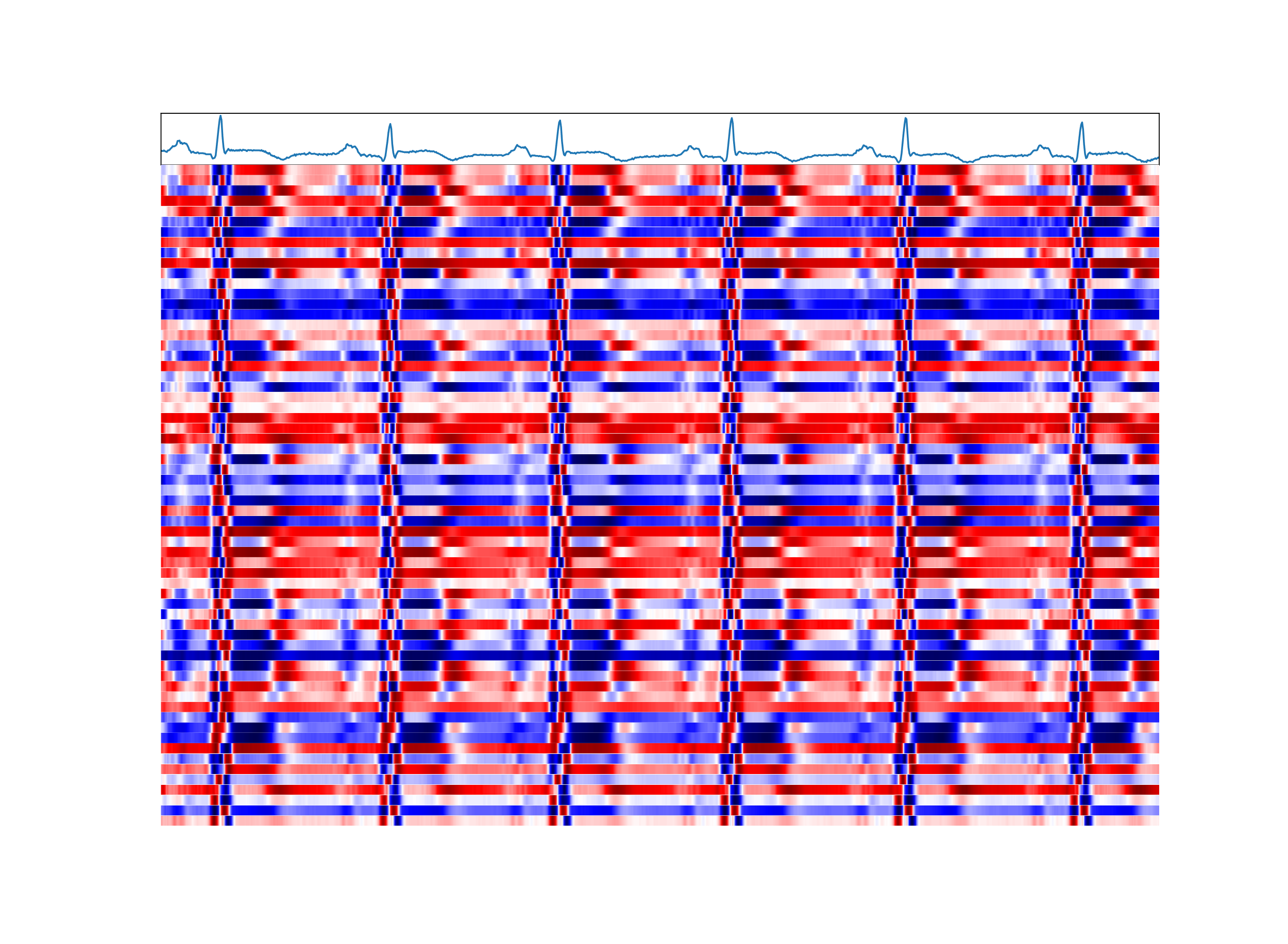


**(b)**


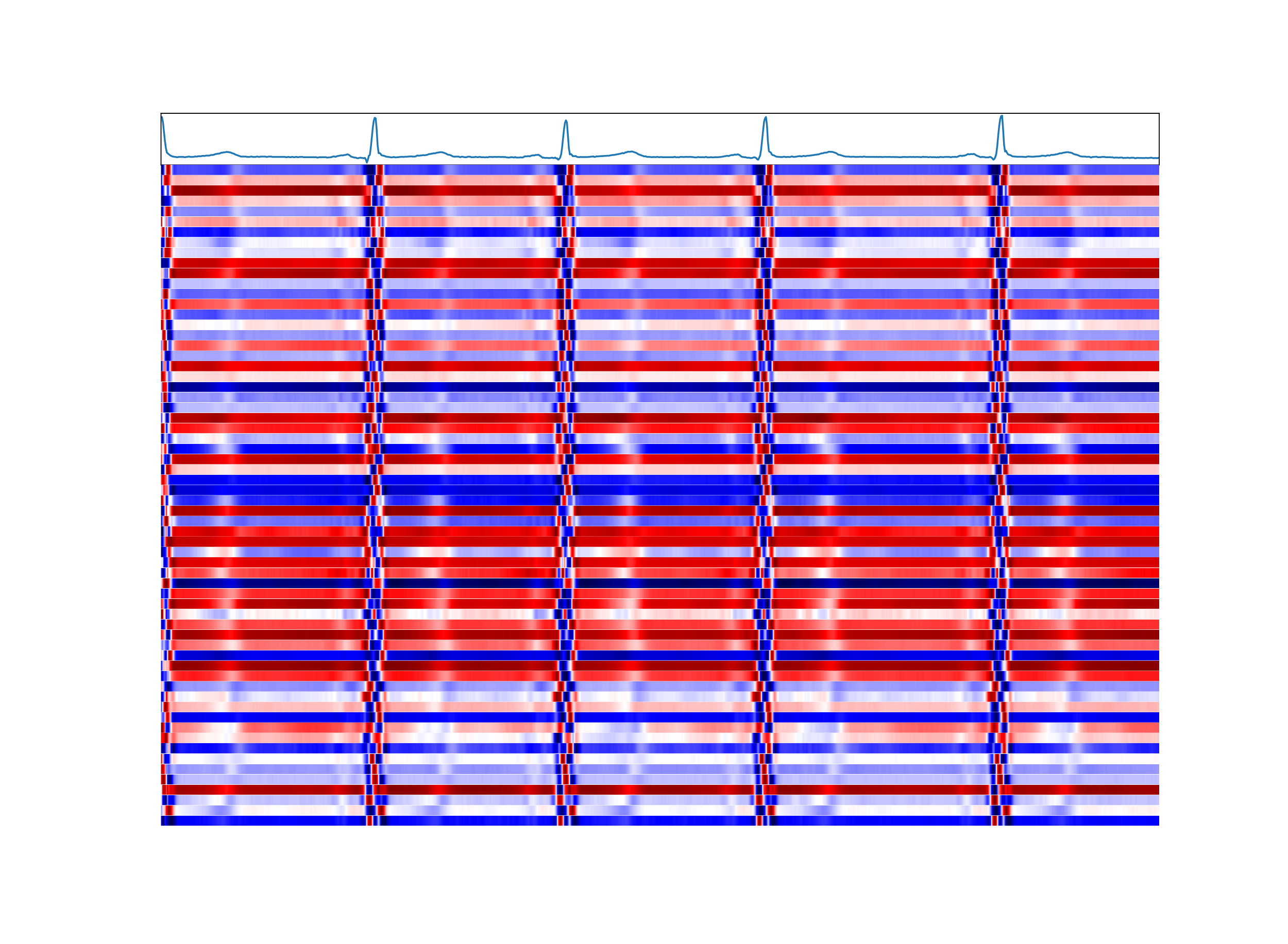


**Supplementary Figure S2:** Full representative saliency maps for the troponin discrimination network trained at a cutoff of 0.02. (a) Positive class (TnI of 50). (b) Positive class (TnI of 23). (c) Positive class (TnI of 7). (d) Negative class (TnI of 0)


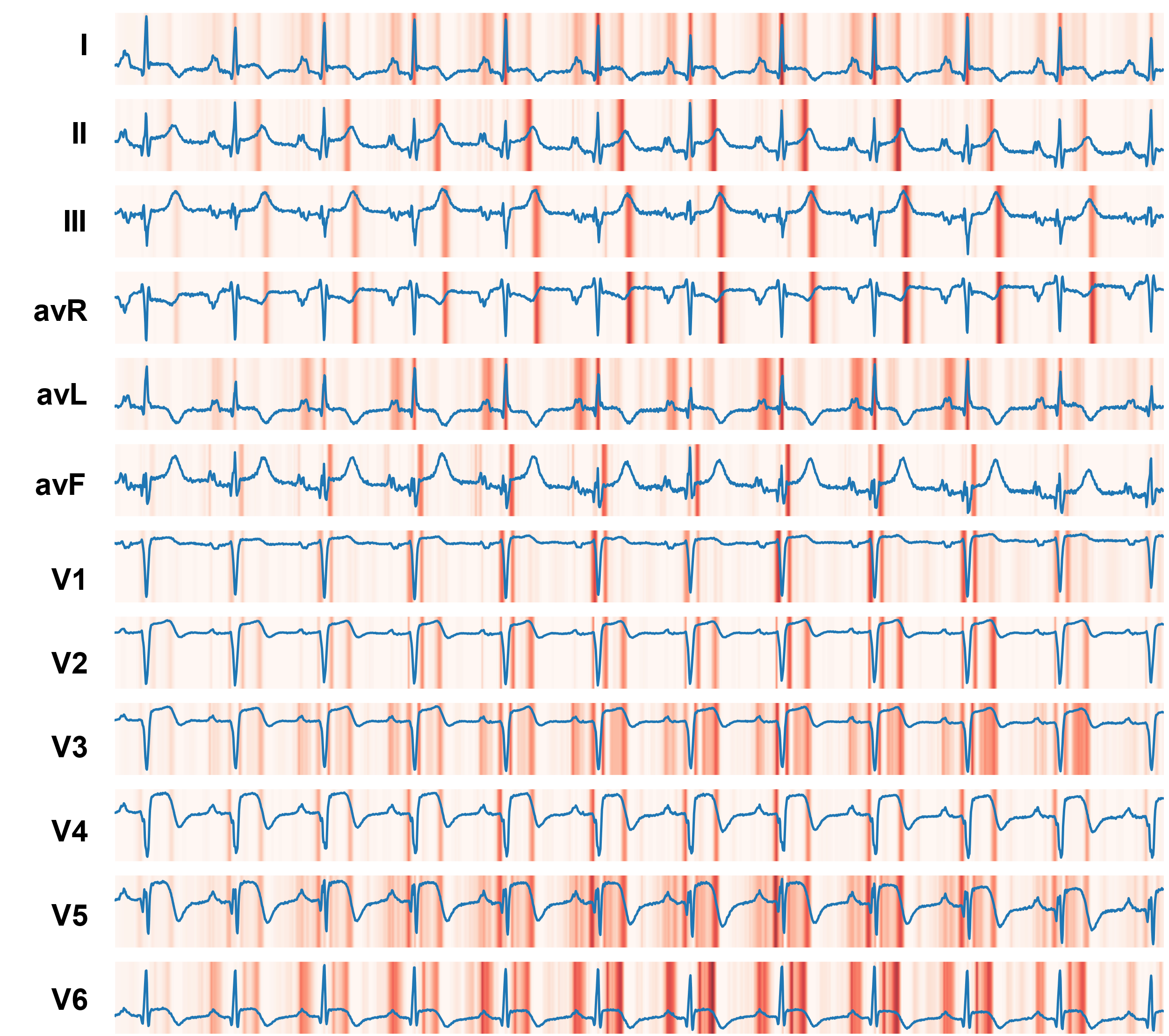


(b)


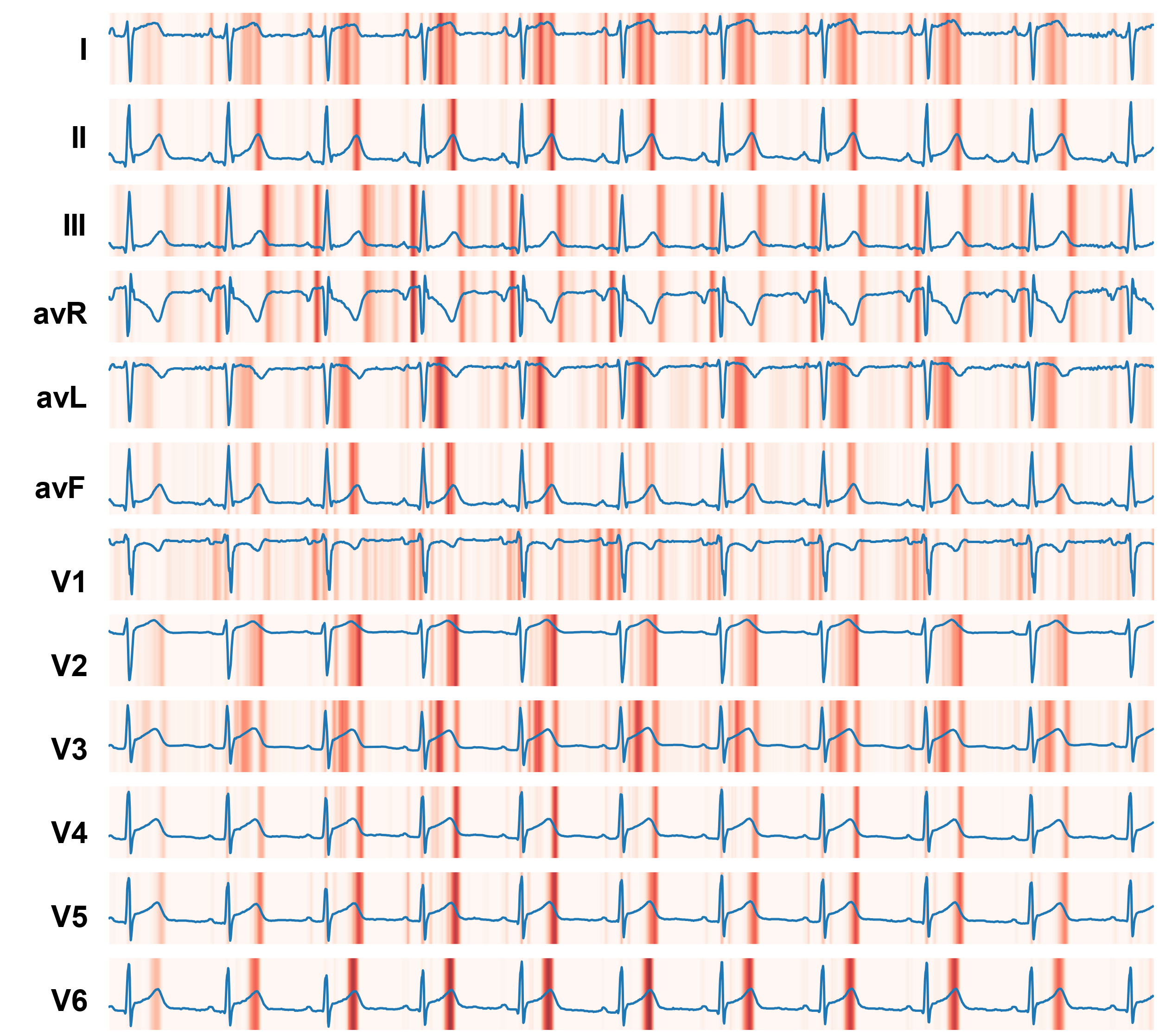


(c)
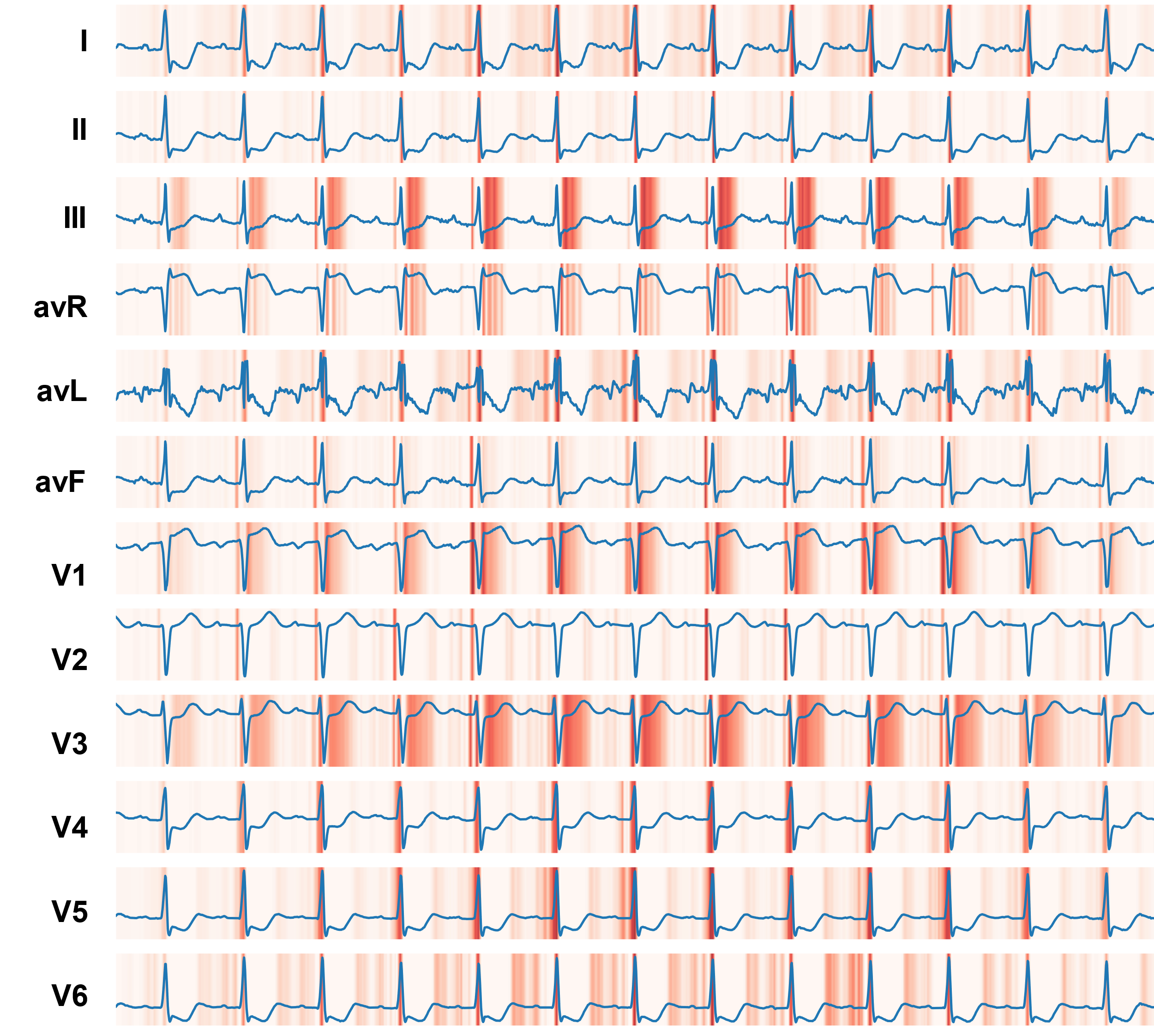


(d)


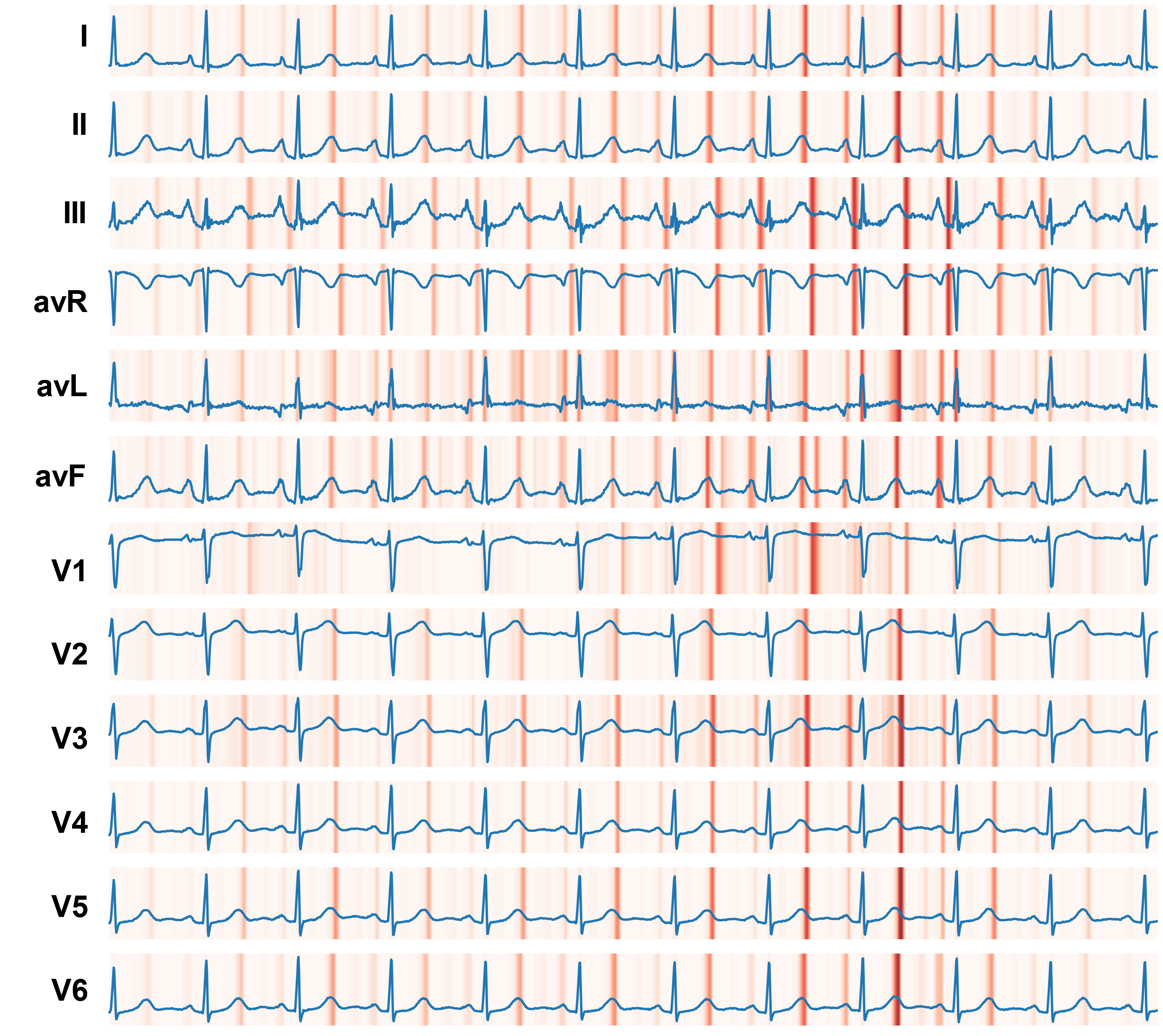

Supplement: Supplementary file 1 — Supplementary Figures. [file 41598_2023_29989_MOESM1_ESM.docx]
